# *In vivo *imaging of lymphocytes in the CNS reveals different behaviour of naïve T cells in health and autoimmunity

**DOI:** 10.1186/1742-2094-8-131

**Published:** 2011-10-06

**Authors:** Josephine Herz, Magdalena Paterka, Raluca A Niesner, Alexander U Brandt, Volker Siffrin, Tina Leuenberger, Jerome Birkenstock, Agata Mossakowski, Robert Glumm, Frauke Zipp, Helena Radbruch

**Affiliations:** 1 Klinik für Neurologie, Universitätsklinik Essen, Hufelandstr. 55, 45122 Essen, Germany; 2 Universiätsmedizin Mainz, Klinik für Neurologie, Langenbeckstr 1, 55131 Mainz, Germany; 3 Deutsches Rheuma-Forschungszentrum (DRFZ), Berlin, Charitéplatz 1, 10117 Berlin, Germany; 4 gfnmediber GmbH, Sophie Charlottenstr 92-94, 14059 Berlin, Germany; 5 Experimental and Clinical Research Center, a joint cooperation between the Charité Medical Faculty and the Max-Delbrück Center for Molecular Medicine, Berlin, Germany, Robert-Rössle Str 10 13092 Berlin, Germany; 6 Labor für Molekulare Psychiatrie, Charité and Berlin-Brandenburg School for Regenerative Therapies (BSRT), Charité Universitätsmedizin Berlin, Charitéplatz 1, 10117 Berlin, Germany; 7 Max-Delbrueck Center for Molecular Medicine Berlin-Buch, Robert-Rössle Str 10 13092 Berlin, Germany

**Keywords:** naïve, T-cell, migration, EAE, second harmonic generation

## Abstract

**Background:**

Two-photon laser scanning microscopy (TPLSM) has become a powerful tool in the visualization of immune cell dynamics and cellular communication within the complex biological networks of the inflamed central nervous system (CNS). Whereas many previous studies mainly focused on the role of effector or effector memory T cells, the role of naïve T cells as possible key players in immune regulation directly in the CNS is still highly debated.

**Methods:**

We applied *ex vivo *and intravital TPLSM to investigate migratory pathways of naïve T cells in the inflamed and non-inflamed CNS. MACS-sorted naïve CD4+ T cells were either applied on healthy CNS slices or intravenously injected into RAG1 -/- mice, which were affected by experimental autoimmune encephalomyelitis (EAE). We further checked for the generation of second harmonic generation (SHG) signals produced by extracellular matrix (ECM) structures.

**Results:**

By applying TPLSM on living brain slices we could show that the migratory capacity of activated CD4+ T cells is not strongly influenced by antigen specificity and is independent of regulatory or effector T cell phenotype. Naïve T cells, however, cannot find sufficient migratory signals in healthy, non-inflamed CNS parenchyma since they only showed stationary behaviour in this context. This is in contrast to the high motility of naïve CD4+ T cells in lymphoid organs. We observed a highly motile migration pattern for naïve T cells as compared to effector CD4+ T cells in inflamed brain tissue of living EAE-affected mice. Interestingly, in the inflamed CNS we could detect reticular structures by their SHG signal which partially co-localises with naïve CD4+ T cell tracks.

**Conclusions:**

The activation status rather than antigen specificity or regulatory phenotype is the central requirement for CD4+ T cell migration within healthy CNS tissue. However, under inflammatory conditions naïve CD4+ T cells can get access to CNS parenchyma and partially migrate along inflammation-induced extracellular SHG structures, which are similar to those seen in lymphoid organs. These SHG structures apparently provide essential migratory signals for naïve CD4+ T cells within the diseased CNS.

## 1. Background

In the last 10 years, two-photon laser scanning microscopy (TPLSM) has revealed the dynamic nature of immune cells within living lymphoid and target organs [1-6]. This has led not only to a better understanding of generation and priming of many immune cells, but also of the basics of immune regulation.

Using TPLSM, we previously showed that activated CD4+ effector T cells are attracted to the CNS's perivascular space and reveal a CXCR4 dependent and vessel-associated migration pattern, suggesting this compartment is highly relevant for autoimmunity and immunoregulation [3,7]. While previous studies of ours as well as other studies mainly concentrated on T cells in their effector or effector-memory state, in the current study we focused on the behaviour of naïve and regulatory T cells.

Activated and memory T cells express adhesion molecules, chemokine receptors and integrins that enable them to cross the blood brain barrier to carry out immune surveillance of the CNS [7]. Adversely, naïve T cells which do not express essential proteolytic enzymes (e.g. matrix metalloproteinases) and adhesion ligands (e.g. LFA-1 and VLA-4), were thought to circulate only between the blood, lymph and secondary lymphoid organs. However, flow cytometry experiments showed that naïve T cells can indeed be found in the healthy, non-inflamed CNS [8,9]. This is also the case for other non-lymphoid tissues including the pancreas, intestine, lung, liver, kidney, skin and testis, where it is thought that this circulation is part of the normal migratory behaviour of naïve T cells [8]. During CNS- inflammation adhesion ligands (i.e. ICAM-1 and VCAM) facilitate T cell recruitment to the CNS [10-12], and they only get activated when they encounter their antigen in the CNS [13].

Naïve T cells could therefore be potential players in CNS immunoregulation and fulfil an important role both in physiological immune surveillance and in pathological autoimmune processes, such as those which occur during Multiple Sclerosis or experimental autoimmune encephalomyelitis (EAE). However, this raises the question how these T cells can migrate through structures of normal tissue in the absence of inflammatory conditions. This is particularly unclear in the CNS in which there is an intricate architecture of neurons, myelinated axons, oligodendrocytes, astrocytes and microglia. Furthermore, the CNS includes a highly complex, extracellular matrix which provides both structural stability and supports homeostasis of the cellular components. Conventional histological examinations offer insights into these questions, but lack the capacity to directly address kinetics, dynamics and functional relevance of T cell trafficking.

Hence, in the current study we applied intravital TPLSM to investigate migratory pathways of naïve T cells in healthy and inflamed CNS.

## 2. Methods

### 2.1. Mice

C57BL/6 mice and C57BL/6 pups were purchased from Charles River (Germany). *β-actin-EGFP *transgenic C57BL/6 (003291, C57BL/6-Tg(ACTB-EGFP)1Osb/J), *OT-2 *(C57BL/6-Tg(TcraTcrb)425Cbn) transgenic mice and *RAG1 -/- *transgenic C57Bl/6 (002216, B6.129S7-Rag1tm1Mom/J) were originally obtained from The Jackson Laboratory (Maine, USA), bred under SPF conditions at the central animal facility of the Charité - University Medicine Berlin (FEM), and kept in-house for experiments in IVC (individually ventilated cages).

C57BL/6 *OT2 *mice, C57BL/6 *2d2 TCR *[14] transgenic mice (kindly provided by A. Waismann), C57BL/6 *Rosa26 tdRFP ("ΔNeo-flip")*mice [15], obtained from H.J. Fehling, or C57BL/6 *β-actin-EGFP *transgenic mice were intercrossed to generate C57BL/6 *OT2 tdRFP*, C57BL/6 *2d2 tdRFP *mice and C57BL/6 *OT2 EGFP *mice, respectively. C57BL/6 *Thy1-21 EGFP *[16] mice were kindly provided by P. Caroni, C57BL/6 *Thy1.1 CerTN L15 *mice were kindly provided by O.Griesbeck [17] C57BL/6 *Foxp3 EGFP *mice were kindly provided by B. and M. Malissen.

All animal experiments were conducted according to the German Animal Protection Law, were approved by the appropriate state committees for animal welfare (LAGeSo, Landesamt für Gesundheit und Soziales) and were performed in accordance with current guidelines and regulations.

### 2.2. Cell culture

Mice (6-10 weeks old) were sacrificed and spleen cells were isolated as described before [3]. For OT-2 Th2 cells splenocytes were cultured in 3 × 10^6^/ml cell culture medium (RPMI 1640 supplemented with 2 mM L-glutamine, 100 U/ml penicillin, 100 μg/ml streptomycin, and 10% fetal calf serum), and stimulated with the respective ovalbumin (OVA) peptide (0.3 μM OVA_323-339; _Pepceuticals, UK). Differentiation towards a Th2 phenotype of OT-2 cells was achieved by the addition of 200U/ml IL-4, 5 μg/ml anti-IL-12 (C17.8) and anti-IFN-γ (AN18.17.24). C57BL/6 Th1 cells were generated from CD4+ sorted spleen and lymph node cells (MACS^®^) of C57BL/6 mice by polyclonal activation by 1 μg/ml plate-bound antiCD3/antiCD28 in the presence of 5 ng/ml IL-12 and 5 μg/ml anti-IL-4 (11B11). Antigen specific and polyclonal effector T cells were restimulated every 7 days by irradiated syngenic CD90-depleted (MACS^®^) APCs or antiCD3/antiCD28, respectively. C57BL/6 Foxp3 expressing regulatory T cells were isolated either by MACS^®^sort for CD4, CD25 and CD62L or by FACS for Foxp3EGFP and CD62L. Cells were polyclonally activated by plate-bound antiCD3/antiCD28 (3/10) in round bottom 96 well plates in the presence of 2000 U IL-2. IL-10 producing Ovalbumin- or MOG-specific regulatory T cells were generated according to the protocol by Barrat et al.[18]. Briefly, naïve CD4+CD62L+T cells were stimulated with irradiated syngenic CD90depleted splenic APCs (MACS^®^) and 0.3 μM OVA_323-339 _or 12,5 μg/ml MOG_35-55 _peptide in the presence of 40 nM Vitamin D3 and 100 nM Dexamethasone and 5 μg/ml anti-IL-12 (C17.8), anti-IFN-γ (AN18.17.24) and anti-IL 4 (11B11). Restimulation was done on day 7 with anti-CD3/anti-CD28. All analysed T cell subsets were stimulated for 3-7 days prior to hippocampal brain slice co-culture experiments. For characterization of T cell phenotypes intracellular staining was performed on short term stimulated (4 h antiCD3/antiCD28) T cells on day 4-7 in order to analyse cytokine and Foxp3 expression by flow cytometry.

For T cells of naïve phenotype spleen and lymph node cells of OT2, OT2 EGFP or OT2 tdRFP mice were MACS^®^-sorted for CD4 and CD62L and used for i.v. injection for lymph node and intravital imaging of the brain stem or for application onto hippocampal brain slices.

### 2.3. Experimental Autoimmune Encephalomyelitis

For adoptive transfer EAE, spleen cells from C57BL/6 *2d2.tdRFP *or C57BL/6 *2d2.EGFP *mice were isolated and stimulated as previously described [19]. Three days after the second restimulation, 5 × 10^6 ^2d2.tdRF TH17 or 2d2.EGFP TH17 cells were transferred intravenously into C57BL/6 *RAG1^-/-^*. Cytokine expression was checked on restimulation and before transfer. Mice were checked for clinical symptoms daily and signs of conventional EAE were translated into clinical score as follows: 0, no detectable signs of EAE; 0.5, tail weakness; 1, complete tail paralysis; 2, partial hind limb paralysis; 2.5, unilateral complete hind limb paralysis; 3, complete bilateral hind limb paralysis; 3.5, complete hind limb paralysis and partial forelimb paralysis; 4, total paralysis of forelimbs and hind limbs (mice with a score above 3.5 to be killed); 5, death. The severity of atypical EAE was translated as: grade 1, tail paralysis, hunched appearance; grade 2, ataxia, scruffy coat; grade 3, head tilt, hypersensitivity, spasticity or knuckling; grade 4, severe proprioception defects; grade 5, moribund.

For intravital imaging of naïve T cells MACS^®^-sorted naïve OT2 EGFP or OT2 tdRFP cells were co-transferred intravenously 12-24 hours before TPLSM or were locally applied on the imaging field at the peak of disease (score 2.5-3.5).

### 2.4. Two-photon laser scanning microscopy

T cells and vessels were visualised by a two-photon laser scanning system (LaVision BioTec, Bielefeld) as described before [20] with dual NIR (850 nm) and IR (1,110 nm) excitation. XYZ-stacks were typically collected within a scan field of 300 × 300 μm at 512 × 512 pixel resolution and a z-plane distance of 2 μm at a frequency of 400 or 800 Hz. Applied laser powers ranged from 2-6 mW at the specimen's surface.

### 2.5. Brain slice T cell co-culture

Preparation of brain slices was performed as previously described [3] and allowed to recover for at least 45 min at room temperature prior to transfer to a heated and with aerated artificial cerebrospinal fluid (ACSF) perfused Luigs & Neumann slice chamber (37°C). T cells, if not expressing EGFP or tdRFP, were stained with celltracker Orange CMTMR (Invitrogen, Germany), pipetted upon the slice and allowed to invade the slice for about 30 - 60 min before image acquisition.

### 2.6. Anaesthesia and preparation of imaging field for intravital imaging

Mice were anaesthetized using 1.5% isoflurane (Abott) in oxygen/nitrous oxide (1:2) with a facemask. They were then tracheotomized and continuously respirated with a Harvard Apparatus Advanced Safety Respirator (Hugo Sachs, Germany). For the brainstem imaging, the anaesthetized animal was transferred to a custom-built microscopy table and fixed in a hanging position. The preparation of the imaging field was performed as previously described [3].

### 2.7. Lymph node preparation for TPLSM

Lymph node preparation was performed as previously described [20]. In brief, 8-96 hours after intravenous injection of naïve OT2 EGFP or OT2 tdRFP T cells, popliteal lymph nodes were removed, glued onto a coverslip and placed into a heated chamber at 36°C, superfused RPMI medium without phenol red bubbled with 95%O2/5%CO2. Naïve T cell migration was visualised up to 130 μm beneath the surface using the same TPLSM setup as described above.

### 2.8. Data analysis

Cell recognition, movement tracking and 3D presentation were performed with Volocity (Improvision, Germany) and tracks with durations > 5 min. were included in the analysis. Average cell velocity was calculated with Volocity. The co-localisation area was calculated with Volocity as previously described [19]. To discriminate short/transient (random) contacts from long-lasting (most probably non-random) interactions, we defined contacts which last longer than 5 min as 'long-lasting'. These interactions were further differentiated into slow/static (velocity < 0.04 μm/s) and dynamic (velocity > 0.04 μm/s) contacts. In order to exclude the SHG signal which is detected with the same detector as EGFP in our setup, we increased the signal threshold so that only EGFP expressing cells (without any SHG signal) were automatically identified and tracked by the software. For angle comparison between cell trajectories and vessel structures and for vessel distance measurements at the end of the track, cell-tracking data from Volocity was further processed in MATLAB as described previously [3]. For each track, a vector between origin and endpoint was calculated in a two-dimensional angle and the distance between the cell-tracking vector and the vector representing the vessel was calculated using standard linear algebra. Statistical analysis was carried out with SPSS (SPSS, Germany) and graphical presentation with GraphPad Prism 4 (Graphpad Software, USA). Results are shown as individual data points; in addition, mean ± SD summarize collective data from performed experiments. To test for statistical significance of differences between two T cell subpopulations, the non-parametric Mann-Whitney-test was performed for more than two groups Kruskal Wallis test with Dunn's multiple comparison test as post test was performed.

## 3. Results

### 3.1. Intravital imaging of the inflamed CNS of living mice reveals distinct migration pattern of naïve T lymphocytes

**Figure 1:**
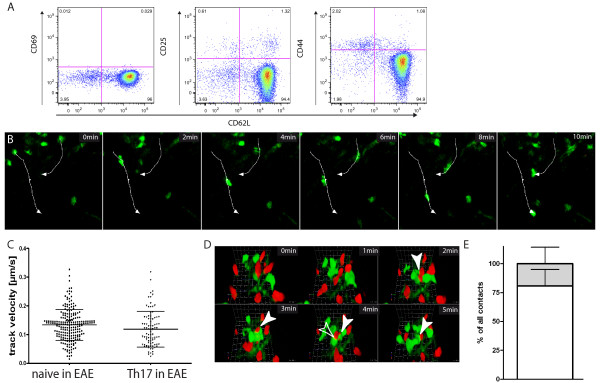
**Intravital imaging reveals high motility of naïve CD4+ T cells in the inflamed CNS**. Naïve OT2 EGFP T cells were intravenously injected into EAE affected mice at the peak of disease (clinical score 2.5) or locally applied onto the imaging field. Intravital TPLSM on the brain stem of these mice was performed 12 - 24 hours after naïve T cell injection or 30 minutes after local application. Adoptive EAE was induced by transfer of *in vitro *generated encephalitogenic 2d2 Th17 T cells into *C57BL/6 RAG1-/- *mice. **(A) **T cell phenotype of MACS isolated naïve T cells was confirmed by FACS analysis prior to experiments. Surface antigen expression of CD62L, CD25, CD69 and CD44 was determined on CD4+ lymphocytes. **(B) **A representative time lapse series derived from intravital TPLSM demonstrates rapid movement of naïve T cells deep in CNS tissue (100-150 μm). Two cell tracks are shown exemplarily by white arrows. For further details see also Additional File 1 (Scale bar: 10 μm.). **(C) **Cell track velocities of naïve OT2 (N = 212) and effector 2d2 Th17 (N = 87) cells at the peak of disease in the inflamed CNS were quantified. The mean track velocities from 4 independent experiments are shown (± SD). **(D) **Contacts (arrowheads) between encephalitogenic 2d2 Th17 effector T cells (EGFP,green) and naïve OT2 (tdRFP red) could be observed during intravital TPLSM, as revealed by the reconstructed 3D time lapse series (80-110 μm). These interactions were mainly short and random like (open arrowhead) although some static long-lasting contacts (filled arrowhead) could be also detected. **(E) **To quantify effector-naïve T cell interactions we analyse the co-localisation area of EGFP and tdRFP as previously described [19]. We discriminated short (random) contacts (< 5 min) from long-lasting (most probably non-random) interactions (≥ 5 min) and observed that 81% ± 14 formed short interactions with effector T cells (white bar) and 19% ± 14 (gray bar) formed long-lasting interactions. Data are shwon as percentage of all contacts from two independent experiments (± SD).

To observe naïve T cell migration under inflammatory circumstances in the CNS, we first performed flow cytometry analysis on brain-derived immune cells after transfer of naïve T cells, which are characterized by high CD62L and low CD69, CD25 and CD44 expression (Figure 1A), into EAE affected Rag 1-/- mice. For disease induction we transferred *in vitro *generated encephalitogenic 2d2 CD4+ TH17 cells (with transgenic T cell receptor specific for Myelin Oligodendrocyte Glycoprotein (MOG)). Some of the injected naïve T cells could be detected in the CNS but the cell number was too low to analyse activation markers and CD62L expression (data not shown). Therefore we used intravital TPLSM in the brainstem to record their behaviour. Using this approach it was possible to find naïve T cells up to 150 μm deep in the CNS parenchyma in EAE and to track their migration (Figure 1B). We used T cells with a transgenic T cell receptor specific for Ovalbumin (OVA) to limit antigen specific activation on their way to and within the CNS. To further minimize activation, our timepoint of investigation was shortly after transfer (12 h for IV and 30 min for local application). Whereas we previously observed no lymphocytes in healthy mice deep in the CNS parenchyma we could detect some sporadic cells in the perivascular space [3]. However, during EAE naïve T cells were able to invade the parenchyma after local application on the brainstem or to cross the corrupted blood brain barrier after IV transfer and to move rapidly (Figure 1B, Additional File 1) resulting in a mean track velocity of 0.13 ± 0.05 μm/sec (± SD; N = 212) which was similar to that of encephalitogenic 2d2 CD4+ TH17 effector cells with 0.12 ± 0.06 μm/sec (±SD; N = 87) (Figure 1C). The dynamic interaction between both cell types was examined by co-localisation analysis as previously described [19]. We discriminated between short (random) contacts and long-lasting (most probably non-random). On their way through the parenchyma 19% of all contacts were of long-lasting nature whereas the majority (81 ± 14%) of the naïve-effector T cell interactions was short and random (Figure 1D/E).

### 3.2. Naïve CD4+ T cells do not get sufficient migratory signals in non-inflamed CNS tissue

**Figure 2:**
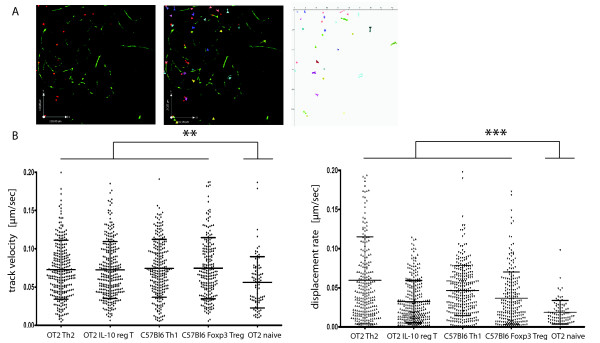
**Naïve CD4+ T cells hardly infiltrate non-inflamed CNS tissue**. MACS isolated CMTMR stained naïve T cells were co-incubated with living hippocampal brain slices in order to visualise their migratory capacity. **(A) **3D time lapse TPLSM revealed a nearly stationary behaviour of naïve T lymphocytes. Cell tracking analysis of individual T cells confirmed these qualitative observations since the corresponding trajectory vectors display almost no displacement of naïve T lymphocytes. (Scale bar: 110 μm). **(B) **Track velocities and displacement rates for naïve T cells are compared with different activated T cell subsets (OT-2 Th2, OT-2/2d2 IL-10 reg T, C57BL6 Th1, C57BL6 Foxp3 Treg) using cell tracking analysis for individual cells of 2-6 independent experiments (± SD). **p < 0.01, ***p < 0.001, Kruskal Wallis Test.

As it was not possible to investigate the behaviour of naïve CD4+ T cells in healthy living anaesthetized mice due to the low frequency of lymphocytes after IV transfer [21], we circumvented the blood brain barrier and applied naive CD4 T cells on the brain stem of healthy living anaesthetized mice. The cells remained stucked on the surface or were flushed away with the perfusion (Additional File 2). We further co-incubated naïve T cells with brain tissue cultured in artificial cerebrospinal fluid (ACSF). These CNS slices have been shown to retain a middle layer which consists of intact *in vivo*-like CNS in murine brain cultures [22]. Naïve CD4+ T cells were labeled with the Celltracker Orange 5-(and-6)-(4-chloromethyl(benzoyl)amino) tetramethylrhodamine (CMTMR) if they were not transgenic for tdRFP or EGFP, and consecutively co-incubated with a living brain slice in which the vasculature had been highlighted by previous IV injection of FITC- or rhodamine-dextran. Independent of the need to transmigrate through the blood brain barrier, these cells showed a nearly stationary behaviour. The cell tracks were short and the cells seemed more or less trapped in their position on the slice surface after application on the non-inflamed CNS tissue (Figure 2A). This was quantified by a significantly lower mean track velocity with 0.056 ± 0.03 μm/sec (± SD; N = 86) as compared to activated CD4+ T cells (**p < 0.01). Furthermore the significantly reduced displacement rate of 0.019 ± 0.015 μm/sec (± SD) shows that they move on their place and do not invade the parenchyma (***p < 0.01) (Figure 2B).

### 3.3. Activated T helper cells infiltrate non-inflamed brain tissue independent of antigen specificity and phenotype and show a perivascular compartmentalisation

**Figure 3:**
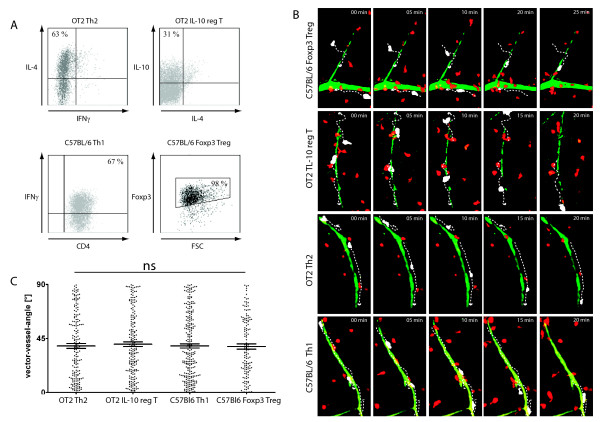
**Activated regulatory and effector CD4+ T cells move rapidly in healthy CNS tissue independent of antigen specificity**. T cell brain slice co-culture experiments were performed for activated antigen-specific IL-10 producing regulatory and effector CD4+ TH2 cells as well as for polyclonally activated Foxp3 regulatory and effector CD4+ TH1 cells. **(A) **T cell subsets were characterized by intracellular staining of typically expressed molecules for each cell type. For detection of interleukins, T cells were stimulated by aCD3/aCD28 (1 μg/ml) prior to staining. **(B) **Movement patterns of polyclonally and antigen-specific activated regulatory and effector T cells were analysed by time lapse TPLSM at 80-130 μm depth after an initial invasion period of 30-45 min. For each series, migratory routes of two exemplary cells (white) are represented by white arrows. **(C) **Vector vessel angels of individual cell tracks of different activated T cell subsets (OT-2 Th2, OT-2/2d2 IL-10 producing regulatory T cells, C57BL6 Th1, C57BL6 Foxp3 Treg) were calculated using cell tracking analysis for individual cells of 2-6 independent experiments per group (± SD). ns: not significant, Kruskal Wallis Test.

The stationary behaviour of naïve CD4+ T cells, as described above, is clearly a contrast to the migration pattern of activated CD4+ TH1 and CD4+ TH2 effector cells. We previously showed that CD4+ antigen specific, highly-activated and differentiated effector T cell lines show a vessel-associated movement pattern [3]. We wanted to check whether antigen specificity or regulatory phenotype would influence T cell migration. Therefore we performed T cell brain slice co-culture experiments as described above exemplarily for activated antigen specific (OT2 or 2d2) IL-10-producing regulatory and antigen specific (OT2) effector CD4+ TH2 cells as well as for polyclonally-activated Foxp3 T regulatory and CD4+ TH1 effector cells (Figure 3A). After an initial invasion period, the potential of tissue penetration was evident for all analysed activated T cell subsets as visualised by time lapse TPLSM. Furthermore, the migration pattern was similar in all investigated CD4+ T cell subtypes. The mean angle between vessel and cell trajectory vectors was similar in all investigated subgroups (Figure 3B, Figure 3C). The vessel-associated movement of CD4+ T cells in contrast to CD8+ T cells [3] seems also to be important for regulatory T cell subtypes independent of antigen specificity and underlines the importance of the perivascular space for immunoregulation and autoimmunity. Activated CD4+ T cells of regulatory phenotypes moved through non-inflamed CNS tissue with 0.07 ± 0.04 μm/s (± SD) for antigen-specific IL-10 producing regulatory T cells (N = 290) and 0.08 ± 0.04 μm/s (± SD) for C57Bl/6 Foxp3 CD4+ T cells (N = 215). Activated effector T cell subsets also revealed comparable mean track velocities, irrespective of antigen specificity and phenotype with 0.07 ± 0.0 μm/s (± SD) for OT2 Th2 (N = 290) and 0.07 ± 0.04 μm/s (± SD) for C57BL/6 Th1 (N = 248) cells (Figure 2B).

### 3.4. Naïve CD4+ T cells reveal high motility in healthy lymphoid organs

**Figure 4:**
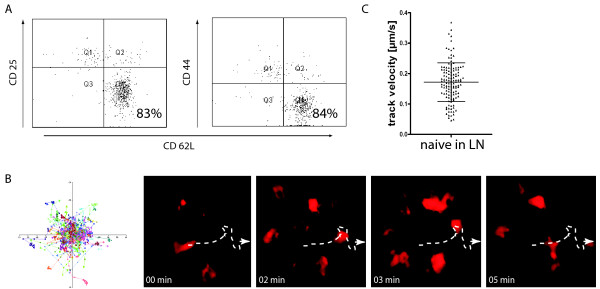
**High motility of naïve CD4+ T cells in healthy lymphoid tissue**. MACS^® ^sorted OT2 tdRFP expressing CD4+ naïve T cells were intravenously injected into healthy *C57BL/6 RAG 1 -/- *mice in order to perform time lapse TPLSM on popliteal lymph nodes at 8 - 96 hours after transfer. **(A) **Injected OT2 tdRFP cells were isolated from lymph nodes of *C57BL/6 OT2 tdRFP *mice and subjected to FACS analysis to measure surface expression of CD62L, CD25, and CD44 at 96 hours after transfer. Dot Plots reveal analysis on CD4+ tdRFP positive lymphocytes. **(B) **High motility of naïve T lymphocytes in lymph nodes is revealed by trajectory vector blots derived from cell tracking analysis of time lapse TPLSM at 24 hours after transfer (left). Each vector represents displacement of individual cells for an imaging period of 30 min (one image every minute) relative to their starting position. A corresponding time lapse series of naïve cell migration is shown on the right. The dashed arrow represents migration of one single cell exemplarily. **(C) **Quantification of naïve CD4+ T cell movement in isolated lymph nodes. The mean track velocity is 0.16 ± 0.06 μm/sec (± SD, N = 73).

As control for the principle migratory capacity of naïve CD4+ T cells we injected MACS^® ^sorted naïve OT2 cells intravenously into healthy mice and tracked their migration pattern in the lymph node (Figure 4). To check whether the cells were activated and primed after transfer we performed FACS analysis on injected cells and observed that these cells retain their CD62L^high ^and CD69^low ^phenotype up to 4 days after transfer (Figure 4A). We performed our experiments within this time frame. Time lapse TPLSM revealed high motility for naïve T cells (Figure 4B). Cell tracking analysis of individual T cells confirmed our qualitative observations, as the mean track velocity with 0.17 ± 0.06 μm/sec (± SD; N = 126) was similar to what was previously described by others [2] (Figure 4C).

### 3.5. Naïve T cells partially migrate along inflammatory induced reticular fibers in vivo

**Figure 5:**
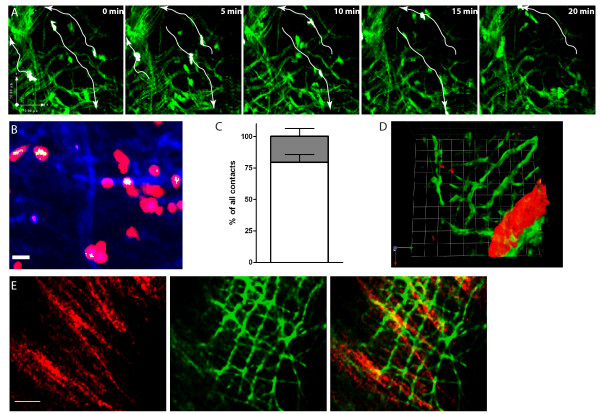
**The role of inflammatory induced reticular fibers in naïve T cell migration in healthy and inflamed CNS tissue**. **(A) **Intravital TPLSM reveals inflammation induced reticular fibers which are visualised by second harmonic generation (SHG) at 1110 nm wavelength in the brain stem of EAE affected mice. Naïve T cells partially migrate along SHG structures as demonstrated by cell tracks represented by white arrows exemplarily. For further details see also Additional File 3. **(B) **To quantify SHG signal and naïve T cell interactions we analyse the co-localisation area of SHG signal (blue) and tdRFP (red). The calculated colocalisation channel (white) was analyzed by determining the duration and the velocity of the tracked contacts (scale bar 10 μm). **(C) **Interaction patterns were differentiated into short (less than 5 min duration) and long-lasting contacts (equal or longer than 5 min duration,), Most of the contacts were short (79% ± 6; N = 164; white bar) but also long-lasting interactions could be detected (20 ± 6%; N = 38; gray bar) and that these long-lasting contacts are due to migration associated co-localisation as their velocity is > 0.04 μm/s. Data from two independent experiments are shown as percentage of all contacts (± SD). **(D) **Visualization of the vasculature by IV injection of Rhodamine-dextrane prior to imaging enabled the visualisation of both, the reticular structures (green) and vessels (red), simultaneously revealing that besides the vessel associated SHG signal a large amount of fibers originates directly from the CNS parenchyma outside the perivascular space. 1 unit = 15 μm **(E) **Intravital TPLSM on brain stem of EAE affected C57BL/6 *Thy1.1 CerTN L15 *mice which express a FRET based calcium sensor. The Citrulin/Cerulean FRET pair is expressed in neurons and is detected by excitation at 850 nm (left, red) whereas SHG signal was generated by a separated excitation at 1110 nm (middle, green), which does not allow to detect either Citrulin or Cerulean. Images are presented in false colors in order to increase the contrast between both separately detected structures. Merge of both images derived from different excitation modes in the same layer demonstrates that the SHG signal (green) is not co-localised to neuronal structures (red). These images are derived form the same layer at a depth of 60 μm below the surface. Scale bar 30 μm.

In lymphoid tissue it is known that naïve T cell motility is directed by fibroblastic reticular cells and ECM structures which can be visualised by second harmonic generation (SHG) [23]. Interestingly, we observed similar SHG signals which were generated by an optical parametric oscillator (OPO) at 1110 nm wavelength in the inflamed CNS of EAE affected mice. Moreover, naïve T cells partially moved along these structures (Figure 5A, Additional File 3). In order to objectify our observation we performed quantitative analysis for naïve T cell - SHG co-localization (Figure 5B/C). This analysis revealed that most of the contacts are short (81 ± 14%) but there is also a population of naive T cells that show long-lasting contacts with the reticular fiber network (19 ± 14%). These interactions were further differentiated into slow/static (velocity < 0.04 μm/s) and dynamic (velocity > 0.04 μm/s) contacts. Interestingly, these long-lasting contacts were due to migration associated co-localisation, since their velocity was > 0.04 μm/s supporting our qualitative observation that a distinct population of naïve T cells partially migrate along these inflammation induced fibers (Figure 5B/C).

The reticular fiber network was observed deep in the parenchyma (up 100 μm) and thus could be distinguished from the arachnoidea covering the surface of the brain stem parenchyma. Moreover, by visualisation of the vasculature by IV Rhodamine-dextrane injection prior to imaging we observed that in addition to the vessel associated collagen network surrounding the very close proximity of the vasculature further fiber tracts are generated deep in the parenchyma itself (Figure 5D).

We could not detect these structures under healthy conditions in the CNS parenchyma (Additional File 2). However, intravital TPLSM on the brain stem of EAE affected C57BL/6 *Thy1.1 CerTN L15 *mice which express a FRET based calcium sensor, i.e. the Citrulin/Cerulean FRET pair (based on YFP/CFP) in neurons and neuronal processes (axons and dendrites) [17], we could distinguish the generated SHG signal due to wavelength specificity of SHG (1110 nm) and separated excitation of Citrulin (850 nm). Using this approach we did not observe any co-localisation with the neuronal structures. In contrast, they appeared to cross the neuronal processes orthogonally, as visualised by merging fluorescence images acquired at both excitation wavelengths (Figure 5E).

## 4. Discussion

Unexpectedly, we observed a highly motile migration pattern for naïve T cells similar to effector T cells in the inflamed CNS of living EAE affected mice. However, we could further show that even though naïve T cells have migratory capacity in lymphoid organs and diseased CNS, they cannot find sufficient migratory signals in healthy non-inflamed CNS parenchyma since they only showed stationary behaviour in this environment. Using different experimental approaches, i.e. locally applied or IV transferred naive T cells, we could detect highly motile cells in the inflamed CNS parenchyma but no migration in healthy tissue supporting the hypothesis that the extend of inflammation is the crucial factor determining the possibility for naive T cells migration in the CNS parenchyma. Interestingly, in the inflamed CNS we could detect reticular structures by their SHG signal which was not detected in the non-inflamed CNS. These structures resemble those present in lymphoid tissues, suggesting that these inflammation-induced structures might provide the chemotactic signals needed for the naïve T cells to migrate within the diseased CNS.

Little is known concerning the behaviour of naïve T cells in non-lymphoid organs under both homeostatic and inflammatory conditions in general. Regarding the CNS even less is known and most of the data are derived from flow cytometry studies of cells isolated from CNS tissue [8,9,13,24,25] or cerebrospinal fluid [26] and give contradictory results. Using an *in vivo *staining method, a recent study did not detect naïve CD4+ T cells in the brain at 4 days after transfer of naïve T cells [24]. In contrast experiments using an inverse transfer model (encephalitogenic cells into OVA specific T cell receptor transgenic mice) show that naïve endogenous T cells occur in parallel with activated encephalitogenic T cells but keep a resting phenotype unless they recognize their antigen in the CNS [13]. Similarly, McMahon et al. could only detect naïve PLP specific T cells in PLP immunized mice in contrast to OVA immunized controls. This supposes that naïve T cells can only enter the CNS if they get locally activated [25].

But in our study we could find naïve OVA specific CD4+ T cells in the inflamed CNS and using intravital TPLSM we could further show for the first time that naïve CD4+ T cells can indeed migrate rapidly through the CNS parenchyma, comparable to the way they move in lymphoid tissue. Due to the low frequency of naïve CD4 T cells compared to effector Th17 cells in the inflamed parenchyma it could be possible that they are not detected by flow cytometry after isolation. Therefore TPLSM could be used as complementary technique to analyse rare cell migration and further allows the investigation of dynamics.

The mean track velocity of naïve T cells was similar to the velocity of Th17 effector T cells. However, it remains unclear what role naïve T-cells play in the CNS. In MS naïve T cells are found in the cerebrospinal fluid during relapses [26] and the experiments from Brabb et al. support a possible function for the development of tolerance [9]: Using MBP (myelin basic protein)-recognizing T-cell receptor transgenic mice, they showed that naïve MBP-specific T cells isolated from the brain did not show any reactivity for MBP *in vitro*. On the other hand, MBP-specific naïve T cells from the periphery did react to MBP. Although we did not investigate the direct impact of naïve CD4+ T cells on function and behaviour of encephalitogenic T cells within the inflamed brain, our observation of direct contacts between naïve T cells and encephalitogenic T cells suggests a possible direct impact. It remains to be clarified whether naïve CD4+ T cells might be of regulatory phenotype and nature in the CNS, and could therefore provide an opportunity for a novel therapeutic approach in MS or other autoimmune diseases.

However, in order to develop new treatment strategies, it is essential to understand how CNS tissue itself regulates naïve T cell trafficking. In lymphoid tissue it is known that T cell motility is directed by fibroblastic reticular cells and ECM structures [23]. Similar reticular structures exist in peripheral tissues such as the liver or skin, but are absent from the non-inflamed brain [27-29]. The source of the ECM structures in the CNS during inflammatory conditions we could detect in this study, however, is unclear. The ECM seems to undergo significant structural changes in the CNS since we could only detect SHG structures under inflammatory conditions. Similarly, Wilson et al. showed a reticular fiber network the produces a SHG signal during *Toxoplasma gondii *infection [29]. SHG generated by excitation at wavelengths of 1000-1500 nm in TPLSM has its origin from highly-ordered, non-centrosymmetric structures such as collagen [30]. As the SHG phenomenon gives no indication of the molecular nature of the structures, it remains unclear which fibers are generating this signal. In the study of Wilson et al. the SHG signal was co-localised with GFAP expressing astrocytes, but with a spatial separation, suggesting that the SHG signal originates from extracellular fiber networks [29]. Furthermore, they found no increased collagen type IV or type I induction but reticular structures by histology which co-localised with CCL21 under inflammatory conditions, suggesting that CCL21 could be one possible candidate, because it is also known that impaired neurons bind CCL21 on their surface [31]. However, as we investigated the co-localisation of neuronal structures and the SHG signal in EAE in transgenic mice expressing EGFP or Citrulin under the neuron specific Thy1 promoter, we could not detect any co-localisation. In contrast, we observed an orthogonal crossing of neuronal structures by the SHG signals, indicating that these structures are not generated by intermediate filaments inside axons or extra cellular matrix co-localised with axons *in vivo*.

Nevertheless, CCL21 bound to these reticular fibers, could be a signal for attracting naïve T cells: Woolf et al. compared soluble vs. matrix-bound CCL21 in an *in vitro *assay and showed that naïve T cells migrated only if the chemokine was matrix-bound [32]. Our experiments are consistent with these results and demonstrate *in vivo *relevance of the described phenomenon. Furthermore, our observations suggest that reticular fibers, analogous to those of the lymph node's ECM, may provide a scaffold that supports T cell migration in the inflamed brain.

## 5. Conclusions

The mechanisms of autoimmunity and tolerance in the brain are still not clear and the role of naïve T cells as possible key players in these complex processes is highly debated [33]. Due to methodological limitations in the past, there remains an ongoing discussion as to what extent naïve T cells enter the healthy non-inflamed versus the inflamed CNS parenchyma and what role they might have. For activated T cells, proteolytic enzymes like metalloproteinases, disintegrins or granzymes are known to enable migration through the inflamed brain tissue [12,34,35]. These enzymes may be even more important if the reticular fiber network is not as abundant, as it is the case under inflammatory conditions where it facilitates immune cell trafficking. In contrast naïve T cells express molecules that are important for migration to secondary lymphoid organs such as L-Selectin and CCR7 for CD4+ T cells [36]. So it was previously thought that they circulate between blood and secondary lymphoid organs exclusively [37,38].

With the opportunity of TPLSM and the availability of various fluorescent protein expressing transgenic mouse models, we are now able to directly visualise the presence and dynamic processes of different T cell subsets deep in CNS tissue in living animals. We show here that the activation status rather than the T cell phenotype and antigen specificity is the central requirement for the migratory capacity of T cells in healthy CNS tissue independent of transmigration-associated molecular interactions at the blood brain barrier. Even though naïve T cells show a high migratory capacity in lymphoid organs, it remains to be seen why they appear in inflamed CNS where they partially migrated along inflammation- induced extracellular SHG structures and which signals might be responsible for the observed high motility in diseased brain tissue of EAE affected mice.

Although further studies are required to define the composition of these reticular networks, targeting these structures might be a novel therapeutic approach to manage T cell-mediated inflammatory central nervous system diseases.

## 6. Competing interests

The authors declare that they have no competing interests.

## 7. Authors' contributions

JH was responsible for executing the research project and, together with HR, writing the manuscript. AUB performed the statistical analysis and assisted in editing the manuscript, RN, VS, TL, MP, JB, AM and RG assisted technically with research and data analysis. FZ contributed to the design of the experiments and assisted in editing the manuscript. HR directed all aspects of this research project including the experimental design, completion of statistical analysis, and writing of the manuscript. All authors read, critically revised and approved the final manuscript.

## Supplementary Material

Additional file 1 **Naïve T cells do not migrate into healthy CNS tissue *in vivo***. Time lapse movie of naïve tdRFP expressing OT2 T cells 30 min after local application on the brain stem of a healthy mouse. Click here for file

Additional file 2 **Movement of naïve T cells in the brain stem during EAE**. Timelapse movie of naïve EGFP expressing OT-2 T cells in the brain stem at peak of disease. Cells were MACS sorted for CD4 and CD62L and transferred intravenously to EAE affected mice at peak of disease. Analysis was performed 12 h after transfer. Click here for file

Additional file 3 **SHG structures are induced in the inflamed CNS and naïve T cells partially migrate along these structures**. Timelapse movie of naïve EGFP expressing OT-2 T cells at peak of disease. Cells were MACS sorted for CD4 and CD62L and transferred intravenously to EAE affected mice at peak of disease. Analysis was performed 12 h after transfer. Second harmonic generated signal was also detected in the 535/50 nm PMT and is displayed as a reticular structure. Click here for file
